# The Book World of Medicine and Science

**Published:** 1895-04-27

**Authors:** 


### The Book World of Medicine and Science.

Dissections Illustrated: A Graphic Handbook for Students of Human Anatomy. By C. Gordon Brodie, F.R.C.S., late Senior Demonstrator of Anatomy, Middlesex Hospital Medical School; Assistant Surgeon North-West London Hospital, with plates drawn and lithographed by Percy Highley. Part IV.—The Abdomen. (Whittaker and Co., London. 1895.)

This completes Mr. Brodie's "Atlas of Dissections." The part contains sixteen coloured plates, and thirteen diagrams. Two of the plates depicting the front of the abdomen are double, and they are all drawn to scale two-thirds of the natural size. The dissections illustrated are those commonly made by students, and the plates are very faithful representations of the actual facts, although in some instances the colouring is too bright and the work deficient in fineness. The handbook, if wisely used, is capable of being of great value to students and practitioners. If employed as a substitute for study of the actual parts it will be an evil, but if employed as an adjunct and help to patient work in the dissecting-room—and this is the author's intention and desire—it will be productive only of good. It must not be forgotten that the atlas has been produced at a modest cost, and of a handy size—both of these are points of great practical importance too often lost sight of. They, of course, introduce difficulties of their own, and they have been very well overcome by Mr. Gordon Brodie and Mr. Highley.

Diseases of the Breast: Their Pathology and Treatment, with Special Reference to Cancer. By W. Roger Williams, F.R.C.S. (London: John Bale and Sons. 1894. pp. 572. Price £1 1s.)

This is a book highly to be commended as giving a careful and reliable account of the diseases of the breast. Special regard is paid to cancer, a subject to which nearly 300 pages are devoted; and, in fact, but for the announcement on the title page that cancer is a matter specially referred to, one might consider the work unduly weighted, and its balance somewhat disturbed, by the minuteness with which all questions pertaining to this subject are entered into. Not that the value of the book is in any way lessened thereby, for the importance of cancer far outweighs that of the other diseases of the breast. The author estimates that there cannot be less than 10,000 women now suffering from cancer of the breast in England and Wales, and no one who understands what is meant by such a sea of misery will think any care too great if so be it can be lessened by better knowledge. After a description of the histology of the breast, both when quiescent and during lactation, it is shown that the pathological neoplastic processes are explicable as aberrant repetitions of those of normal development, and on these lines the various growths occurring in the breast are classified. The preponderance of cancer over other neoplasms in the breast is well shown by the fact that of 2,422 cases of mammary neoplasms consecutively under treatment at Middlesex, University College, St. Thomas's, and St. Bartholomew's Hospitals, 1,879 were cancers. The question is fully discussed whether cancers arise through a modification of the formative procees or are the outcome of the inflammatory process, owing to the presence of micro-organisms or some other sources of irritation. The author holds strongly that the microbes have nothing to do with it. "The microbe of cancer has not yet been discovered, because in all probability it does not exist." The author is quite convinced that cancer is increasing in prevalence He advocates free and complete removal by operation, the great point being that care be taken to remove every particle of diseased structure. If this is done, "it is well within the limit to expect complete cures after thorough operation for cancer of the breast, in at least 15 per cent. of all cases." The book is well written, well printed and illustrated, and is a most interesting guide to the study of the diseases of which it treats.

